# Enhancement of Omicron-specific immune responses following bivalent COVID-19 booster vaccination in patients with chronic lymphocytic leukaemia

**DOI:** 10.1038/s41408-023-00940-5

**Published:** 2024-01-25

**Authors:** Thomas Roberts, Grace Uwenedi, Rachel Bruton, Graham McIlroy, Sarah Damery, Panagiota Sylla, Nicola Logan, Sam Scott, May Lau, Ahmed Elzaidi, Siobhan Plass, Soumyajit Mallick, Katie Spencer, Christine Stephens, Christopher Bentley, Guy Pratt, Jianmin Zuo, Shankara Paneesha, Brian Willett, Paul Moss, Helen Parry

**Affiliations:** 1 Institute of Immunology and Immunotherapy, University of Birmingham, Birmingham, B15 2TT UK; 2 University Hospitals Birmingham, Edgbaston, Birmingham, B15 2GW UK; 3 Institute of Applied Health Research, University of Birmingham, Birmingham, B15 2TT UK; 4 MRC-University of Glasgow Centre for Virus Research, University of Glasgow, Glasgow, G61 1QH UK

**Keywords:** Immunological memory, Chronic lymphocytic leukaemia

Cancer patients who are immune suppressed remain at increased risk from COVID-19 and have recently been prioritised for additional booster bivalent vaccines. Despite this, previous studies have shown that humoral and cellular immune responses often fail to reach levels comparable to the general population following booster doses. The emergence of the omicron variant of SARS-CoV-2 has necessitated the deployment of modified bivalent mRNA vaccines that direct the synthesis of spike protein from Omicron in combination with ancestral spike. Bivalent booster vaccines have shown utility in general population studies [1–3] but there is little understanding of their relative immunogenicity in patients with immune suppression.

Chronic lymphocytic leukaemia (CLL) is the most common subtype of leukaemia and is associated with significant immune suppression. Indeed, mortality rates following SARS-CoV-2 infection approached 33% in the pre-vaccine era [4]. Here we studied SARS-CoV-2 immunity in a cohort of 84 patients with CLL who received an original/Omicron BA.1 bivalent vaccine during their booster vaccine regimen. This represented the 6th vaccine for 66% of recipients and the 5th vaccine for 34%, whilst the median time since the previous vaccine was 154 days (IQR 123–215). 57% received the Moderna Spikevax bivalent Original/Omicron BA.1 vaccine whilst 43% received the Pfizer Comirnaty Original/Omicron BA.1 vaccine. Blood samples were taken 10 days (3–28) prior to vaccine delivery (*n* = 50) and/or at 40 days following vaccination brackets (IQR 31–56.5) (*n* = 67).

Antibody responses against ancestral spike protein were initially measured using the Roche Elecsys platform which had been employed throughout this prospective cohort study [5] (methods available in Supplementary materials). Excluding participants with prior infection, 87% (58/67) of patients demonstrated a measurable antibody response compared to 100% within healthy donors. Encouragingly, antibody titres amongst responders were equivalent to those seen within healthy controls, a feature not seen following previous booster vaccines (Fig. 1a). Furthermore, titres were now seen to be equivalent in patients with or without clinical or serological evidence of prior natural SARS-CoV-2 infection and it was noteworthy that responses were also detectable in 91% (10/11) of patients with pan-hypogammaglobulinaemia. Nine patients remained seronegative, confirming prior evidence for a plateau of serological response in patients with CLL, and 6 of these were taking Bruton tyrosine kinase inhibitor medication, which is a highly effective treatment but suppresses humoral immunity. Two patients were untreated whilst one patient had received venetoclax and obinutuzumab therapy (Fig. 1b).

**Fig. 1:**
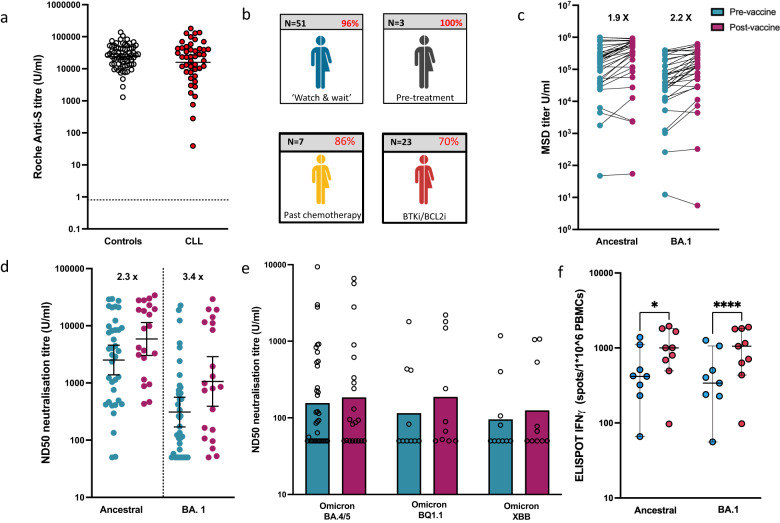
The relative antibody and cellular immune responses following bivalent vaccination in patients with CLL. **a** Spike-specific antibody titre in participants with a positive antibody response following bivalent vaccine dose (*n* = 41) compared to controls (*n* = 70) (*p* = 0.188; Mann–Whitney *U* test). Participants with evidence of natural infection were excluded. The cut-off for a positive response is indicated by the dotted line (Geometric mean and 95% CI shown). **b** Infographic to show percentage with detectable antibody response by disease group characteristic. Those grouped in BTKi/BCL2i represent all donors who have received or continue to take either line of treatment, since January 2021 (*n* = 23). **c** Ancestral B.1 (*n* = 34) and BA.1 (*n* = 33) specific antibody responses before and after bivalent vaccine dose in infection naïve participants (fold change shown). **d** ND50 neutralising antibody titres against viral pseudotypes bearing ancestral or BA.1 Omicron spike glycoproteins pre and post-bivalent vaccine dose amongst 37 infection naïve participants (fold change, GM and 95% CI shown; *p* < 0.0001 Kruskal–Wallis with BA.1 comparison by paired multiple comparison test *p* = 0.059). **e** ND50 neutralising antibody titres against viral pseudotypes for the latest variants of concern (BA.4/5, BQ1.1 and XBB) are shown in 10 infection naïve donors (Geometric mean and 95% CI shown). **f** IFN-gamma ELISpot assay before and after bivalent vaccine dose in infection-naïve CLL. patients following peptide stimulation with either ancestral (*p* = 0.01) or BA.1 Omicron peptide pool (*p* = < 0.0001) (Friedman test Median and IQR shown) (*n* = 9).

We next determined relative antibody generation against ancestral and Omicron spikes to assess the specificity of serological response following bivalent vaccination. The mean antibody titre against the ancestral spike was 2.8 fold higher than against Omicron and is likely to reflect the multiple vaccine exposures to ancestral spike immunogen. However, antibody levels against the ancestral spike increased by 1.9 fold (*p* = 0.14) following the bivalent vaccine compared to a 2.2-fold increase to the Omicron BA.1 variant (*p* = 0.07) (Fig. 1c). We next assessed antibody functional activity by neutralisation of ancestral or Omicron BA.1 spike protein-pseudotyped viruses. Again, although overall neutralising activity was superior against the ancestral variant, relative neutralisation following bivalent vaccination improved only 2.3-fold against the ancestral spike (*p* = 0.23) compared to 3.4-fold against the BA.1 protein (*p* = 0.05) (Fig. 1d). As such, a preferential increment in serological response against Omicron BA.1 was observed following bivalent vaccination. Despite this, the neutralisation of recent Omicron subvariants, including XBB, was limited (1e).

Cellular immunity against SARS-CoV-2 is believed to be important in controlling severe COVID-19 and may be particularly valuable in patients with humoral immune suppression. As such, we next assessed the cellular response against peptides from either ancestral or Omicron spike protein. Robust cellular immunity was observed with a 1.4-fold relative increase in ELISpot response following bivalent vaccination against ancestral spike peptides (*p* = 0.01) compared to 3.1-fold against Omicron peptides (*p* ≦ 0.0001) (Fig. 1f).

Limitations of this study include the small number of patients on BCL2 inhibitor therapy and the lack of neutralisation data against the more recent viral variant BA.2.86. However, our analysis of antibody binding, antibody neutralisation and cellular immunity against the ancestral and Omicron spike show relative enhancement against the Omicron variant following bivalent vaccination which augurs well for responses to future variant-specific vaccines. Despite this, we observed relatively poor neutralisation of Omicron subvariants which supports delivery of updated vaccines based on current circulating variants. A subset of patients with CLL that continue to lack antibody responses, due to ongoing treatment or underlying immune suppression, will also require additional protective approaches such as prophylactic antibody administration in order to minimise the risk of infection.

In conclusion, our data reveal the value of a regime of multiple booster vaccinations for patients with immune suppression and demonstrate encouraging immunogenicity of bivalent vaccines in this vulnerable population.

### Supplementary information

Supplementary materials

## Data Availability

All datasets used during the current study are available from the corresponding author upon reasonable request.
