# Supplementary material for: Enhancement of Omicron-specific immune responses following bivalent COVID-19 booster vaccination in patients with chronic lymphocytic leukaemia
Source: Blood Cancer J. 2024 Jan 25;14(1):22. doi: 10.1038/s41408-023-00940-5 (PMC10810811; doi:10.1038/s41408-023-00940-5)
Supplement: Supplementary file 1 — Supplementary materials [file 41408_2023_940_MOESM1_ESM.docx]

**Methods**

**Study design and participants**

Patients with a diagnosis of CLL or small lymphocytic leukaemia (SLL) were recruited to study with no additional exclusion criteria. Informed consent was obtained by remote consultation and work performed under the CIA UPH IRAS approval (REC 20\NW\0240) from North-West and Preston ethics committee and conducted according to the Declaration of Helsinki. The dates and type of SARS-CoV-2 vaccination were obtained with self-reported information on stage and date of CLL diagnosis and CLL treatment dates. Participant demographics can be found in table 1. Samples were obtained immediately before and 3-4 weeks following the BA.1 bivalent vaccine dose. Healthy donor controls were recruited from local primary care networks.

**Antibody testing**

Roche Elecsys® electrochemiluminescence immunoassay (ECLIA). Using ECLIA, qualitative IgG/A/M Anti-nucleocapsid protein (NP) antibodies specific to SARS-CoV-2 were detected (COV2, Product code: 09203079190); cut-off index value ≥ 1.0 considered positive for anti-nucleocapsid antibodies. Using the quantitative ECLIA assay, anti-spike (S) receptor binding domain antibodies were detected (COV2 S, Product code 09289275190) with values ≥ 0.8 U/ml considered positive. Analysis was performed at UK Health Security Agency, Porton Down, UK.

**MSD testing**

Quantitative IgG antibody titers were measured against Ancestral spike (S) protein and B.1.1.529; BA.1; BA.1.15 using the MSD V-PLEX COVID-19 panel 25 (Cat # K15583U) from Meso Scale Diagnostics, Rockville, MD USA (Appendix). Antigens were spotted at 200−400 μg/mL. Multiplex MSD Assays were performed as per the instructions of the manufacturer. Results were measured in duplicate and averaged. To measure IgG antibodies, 96-well plates were blocked with MSD Blocker A for 30 minutes. Following washing, with washing buffer, the samples were diluted 1:500 in diluent buffer. Reference standards and positive controls and diluted samples were added to the wells. After 2-hour incubation and plates were washed 3x with wash buffer and detection antibody (MSD SULFO-TAG™ Anti-Human IgG Antibody, 1/200) diluted in diluent 100 was added. After 1 hour of incubation at RT, the plates were washed 3x with wash buffer. MSD GOLD™ Read Buffer B was added and plates were read immediately using a MESO TM QuickPlex SQ 120. Text files were then generated from the Methodical Mind software then transferred to the MSD Discovery Workbench (v4.0) software. Data were then converted to AU/ml and exported as .csv files. The values from exported data were then adjusted for any sample dilution.

**Immunoglobulin testing**

Serum Immunoglobulin concentration Quantification of IgG, IgA and IgM was evaluated using the COBAS 6000 (Roche) at the University of Birmingham Clinical Immunology Service.

**Neutralisation assay**

A pseudotyped‑virus neutralisation which has been shown previously to have excellent correlation with live virus neutralisation assay for SARS-CoV-2 was utilised in this work^1^. HEK293, HEK293T and 293-ACE2 cells were maintained in Dulbecco’s modified Eagle’s medium (DMEM) supplemented with 10% foetal bovine serum, 200 mM L-glutamine, 100 μg/ml streptomycin and 100 IU/ml penicillin. HEK293T cells were transfected with the appropriate SARS-CoV-2 spike gene expression vector in conjunction with lentiviral vectors p8.91 and pCSFLW using polyethylenimine(PEI, Polysciences, Warrington, USA). Pseudotype-containing supernatants were harvested 48 hours post-transfection, aliquoted and frozen at -80^o^C prior to use. The SARS-CoV-2 spike glycoprotein expression constructs for ancestral Hu-1, B.1.617.2 and Omicron have been described previously^2^. The BQ1.1 and XBB constructs bore the following mutations relative to the ancestral Hu-1 sequence (GenBank: MN908947): BQ.1.1 - T19I, △24/26, △69/70, G142D, V213G, G339D, R346T, S371F, S373P, S375F, T376A, D405N, R408S, K417N, N440K, K444T, L452R, N460K, S477N, T478K, E484A, F486V, Q498R, N501Y, Y505H, D614G, H655Y, N679K, P681H, N764K, D796Y, Q954H, N969K and XBB - T19I, △24/26, V83A, G142D, △144, H146Q, Q183E, V213E, G339H, R346T, L368I, S371F, S373P, S375F, T376A, D405N, R408S, K417N, N440K, V445P, G446S, N460K, S477N, T478K, E484A, F486S, F490S, Q498R, N501Y, Y505H, D614G, H655Y, N679K, P681H, N764K, D796Y, Q954H, N969K. HIV (SARS-CoV-2). 293-ACE2 target cells were maintained in complete DMEM supplemented with 2 μg/ml puromycin. Neutralising activity in each sample was measured by a serial dilution approach. Each sample was serially diluted in triplicate from 1:50 to 1:36,450 in complete DMEM prior to incubation with approximately 1 Å~ 106 CPS per well of HIV (SARS-CoV-2) pseudotypes, incubated for 1 h, and plated onto 239-ACE2 target cells. Luciferase activity was quantified after 48–72 h by the addition of Steadylite Plus chemiluminescence substrate and analysis on a Perkin Elmer EnSight multimode plate reader (Perkin Elmer, Beaconsfield, UK). Antibody titre was then estimated by interpolating the point at which infectivity had been reduced to 50% of the value for the ‘no serum’ control samples.

**ELISPOT assay**

250,000 PBMCS were incubated overnight with peptide pools containing 15-mer peptides overlapping by 10aa from either SARS-CoV-2Spike S1 or S2 protein domains for the original Wuhan Strain and Omicron variant  (JPT Peptide Technologies (Germany)) ELISPOTs were run in duplicate and results averaged. T cell responses were determined using a Human IFNγ ELISpot PRO kit (Mabtech, Sweden) and plates read using the BioSys Bioreader5000.

**Statistical analysis**

Statistical analysis and sample number utilised in individual experiments are detailed in the figure legends.

**Supplementary reference**

1. Cantoni, D., Wilkie, C., Bentley, E.M., Neto, M.M., Wright, E., Scott, S., et al (2023). Correlation between pseudotyped virus and authentic virus neutralisation assays, a systemic review and meta-analysis of the literature. *Frontiers in Immunology* 14. DOI [10.3389/fimmu.2023.1184362](http://dx.doi.org/10.3389/fimmu.2023.1184362)
2. Willett, B.J., Grove, J., MacLean, O.A. *et al* (2022).SARS-CoV-2 Omicron is an immune escape variant with an altered cell entry pathway. *Nat Microbiol* 7, 1161–1179 (2022). https://doi.org/10.1038/s41564-022-01143-7
